# Diabetes and lipid screening among patients in primary care: A cohort study

**DOI:** 10.1186/1472-6963-8-25

**Published:** 2008-01-30

**Authors:** Sheryl L Rifas-Shiman, John P Forman, Kimberly Lane, Herve Caspard, Matthew W Gillman

**Affiliations:** 1 Obesity Prevention Program, Department of Ambulatory Care and Prevention, Harvard Medical School/Harvard Pilgrim Health Care, 133 Brookline Avenue, 6^th ^Floor, Boston, Massachusetts 02215, USA; 2 Renal Division, Department of Medicine, Brigham and Women's Hospital, 75 Francis Street, Boston, Massachusetts 02115, USA; 3 Department of US Medical Affairs, Sanofi-Aventis, 55 Corporate Drive, Bridgewater, NJ 08807, USA; 4 Department of Nutrition, Harvard School of Public Health, 677 Huntington Avenue, Boston, Massachusetts 02215, USA

## Abstract

**Background:**

Obesity is associated with increased cardiovascular diseases and diabetes mellitus. Guidelines call for intensified glucose and lipid screening among overweight and obese patients. Data on compliance with these guidelines are scarce. The purpose of this study was to assess rates of diabetes and lipid screening in primary care according to demographic variables and weight status.

**Methods:**

Over a 3-year follow-up period, we assessed screening rates for blood glucose, triglycerides, and HDL- and LDL-cholesterol among 5025 patients in primary care. From proportional hazards models we estimated screening rates among low, moderate, high, and very-high risk patients and compared them with recommendations of the American Diabetes Association (ADA), National Cholesterol Education Program (ATP III) and U.S. Preventive Services Task Force (USPSTF).

**Results:**

Mean (SD) age was 47.4 (15.6); 69% were female, 21% were non-white, and 30% of males and 25% of females were obese (BMI ≥ 30 kg/m^2^). For both diabetes and lipid screening, the adjusted hazard was 260–330% higher among ≥65 than <35 year-olds, 50–90% higher in persons with BMI ≥ 35 than <25 kg/m^2^, 10–30% lower for females than males, and not lower among racial/ethnic minorities. Screening rates were at least 80% among very-high risk persons, which we defined as 55–64 years old, BMI ≥ 35 kg/m^2^, non-white, with baseline hypertension. In contrast, high-risk persons who were younger (35–44 years old) and less obese (BMI 30–<35 kg/m^2^) were screened less often (43% for LDL-cholesterol among females to 83% for diabetes among males) even though ADA, ATP III and USPSTF recommend diabetes and lipid screening among them.

**Conclusion:**

Patients with higher BMI or age were more likely to be screened for cardiometabolic risk factors. Women were screened at lower rates than men. Even in a highly structured medical group practice, some obese patients were under-screened for diabetes and dyslipidemia.

## Background

An estimated 97 million adults in the United States are overweight or obese [1]. The presence of overweight and obesity substantially increases the risk of morbidity from several diseases, particularly cardiovascular diseases and diabetes mellitus [2,3].

Clinical guidelines call for intensified diabetes and lipid screening among overweight and obese v. non-overweight persons [4-7] (Table 1). Despite these recommendations few data exist on compliance with them.

**Table 1:** Guidelines for diabetes mellitus and lipid screening.

**Diabetes mellitus screening**
Recommendations of the American Diabetes Association (ADA) [4]
Testing should be considered for those ≥45 years of age and repeated every 3 years, if results are normal.
Testing should be considered younger or more frequently for those who:
*(i) *are African-, Hispanic-, Native-, Asian-, or Pacific Island-American
*(ii) *are overweight (BMI, kg/m^2 ^≥ 25);
*(iii) *have had gestational diabetes or delivered a baby weighing >9 pounds;
*(iv) *have a positive family history of diabetes (parents or siblings);
*(v) *have hypertension (blood pressure ≥ 140/90 mmHg);
*(vi) *have low HDL cholesterol (≤ 35 mg/dl) and/or high triglyceride level (≥ 250 mg/dl);
*(vii) *have had impaired glucose (110 ≤ fasting plasma glucose < 126 mg/dl or 140 ≤ oral glucose tolerance test < 200 mg/dl).
Recommendations of the U.S. Preventive Services Task Force (USPSTF) [5]
The USPSTF recommends screening for type 2 diabetes in adults with hypertension or hyperlipidemia.
**Lipid screening**

Recommendations of the National Cholesterol Education Program (ATP III) [6]
In adults ≥20 years of age, a fasting lipoprotein profile (total cholesterol, LDL and HDL cholesterol) is recommended every 5 years. More frequent measurements are recommended for persons with multiple risk factors or, in those with 0–1 risk factor, if the LDL level is only slightly below the goal level. If the testing opportunity is non-fasting, only the values for total and HDL cholesterol will be usable. In such a case, if total cholesterol ≥200 mg/dL or HDL < 40 mg/dL, a follow-up lipoprotein profile is needed for appropriate management based on LDL.
Recommendations of the U.S. Preventive Services Task Force (USPSTF) [7]
The USPSTF recommends that clinicians screen men aged ≥35 years and women aged ≥45 years and younger adults (men 20–35 and women 20–45 years) if they have other risk factors for coronary heart disease (diabetes, a family history of cardiovascular disease before age 50 years in male relatives or age 60 years in female relatives, a family history suggestive of familial hyper-lipidemia, multiple coronary heart disease risk factors [e.g. tobacco use, hypertension]). The USPSTF recommends that screening for lipid disorders include measurement of total cholesterol and HDL-cholesterol measured on non-fasting or fasting samples. The optimal interval for screening is uncertain. On the basis of other guidelines and expert opinion, reasonable options include every 5 years, shorter intervals for people who have lipid levels close to warranting therapy, and longer intervals for low-risk people who have had low or repeatedly normal lipid levels.

During the years 1998 to 2000, Ealovega et al [8] assessed 3-year diabetes screening rates among 8286 non-diabetic patients, aged ≥45 years, who were members of a health management organization in Michigan. Overall 69% were screened. In multivariate logistic regression analysis, only history of hypertension (OR 3.96 [95% CI 2.53, 6.19]) and history of dyslipidemia (3.96 [2.53, 6.19]) were independent predictors of screening. However, reported prevalence of overweight or obesity in this study was less than 10%, and thus the results may not be generalizable to other populations.

Kern et al [9] assessed 3-year diabetes screening rates among 301 non-diabetic patients at an academic general internal medicine practice in New York City. Overall 78% were screened. In multivariate logistic regression analysis, age (OR 12.36 [95% CI 3.41, 44.79] for ≥45 v. <45 years), sex (0.45 [0.21, 0.93] for female v. male), ethnicity (3.71 [1.68, 8.20] for non-white v. white), family history of diabetes (2.98 [1.12, 7.93]), BMI (1.08 [1.00, 1.18] per unit, kg/m^2^), and number of visits (1.33 [1.12, 1.58] per visit) were independent predictors of screening. However, these results may not be generalizable to non-academic health care settings, the sample size was small, and the authors did not assess screening rates according to body mass index (BMI) level.

In the 2001 Behavioral Risk Factor Surveillance System (BRFSS), 72.5% of participants reported having their blood cholesterol checked in the past 5 years [10]. Unadjusted predictors of cholesterol screening included sex (74.7% among females v. 70.5% among males), race/ethnicity (75.3% among white non-Hispanics v. 64.2% among Hispanics) and age (43.1% for persons 18–24 years to 90.4% for persons age 65–74 years). However, the BRFSS relied on self-reports, which can result in over- or under-estimates of prevalence of screening compared with health care records. Also, that study did not assess screening rates by BMI category or control for confounding variables.

The objective of this study was to examine diabetes and lipid screening rates according to demographic variables and BMI categories in a setting where screening rates are likely to be relatively high.

## Methods

### Participants

The patients for this study were a subset of participants in the HMO Research Network's Center for Education and Research on Therapeutics Patient Safety Cohort Study [11]. Eligibility criteria for this analysis included being a member of both Harvard Pilgrim Health Care (insurance plan) and a multi-site, multi-specialty group practice (Harvard Vanguard Medical Associates), being continuously enrolled throughout 1999, and having a BMI measurement between January 1, 2000 and December 31, 2000.

Using electronic medical records, we identified 13,846 patients who were members of Harvard Pilgrim Health Care and Harvard Vanguard Medical Associates and who had a visit in 2000. Of 13,846 we excluded 6451 patients who did not a BMI measurement between January 1, 2000 to December 31, 2000. Of 7395 patients who met eligibility criteria for this analysis, we excluded 1467 whose membership ended before June 30, 2001 (not enough time to assess follow-up screening) and 536 who had an ICD-9 diagnosis of one of the following medical conditions related to weight loss before June 30, 2001 (to assure the index BMI was not altered by pre-existing illness): AIDS, cancer, malabsorption syndrome, or alcohol use disorder, and 367 who had a cardiovascular event before the index date (congestive heart failure, unstable angina, myocardial infarction, coronary artery bypass grafting, percutaneous coronary intervention, coronary artery disease, ischemic stroke, visceral arterial disease, or peripheral arterial disease). These exclusions left a cohort for analysis of 5025 patients.

Comparison of the 5025 participants in this analysis with 5716 of the 10,741 not included in this analysis showed a higher proportion of females (69% v. 56%), lower proportion of white race (79% v. 83%), slightly lower mean age (46.6 v. 47.6 years) and BMI (27.2 v. 27.5 kg/m^2^), but did not vary by proportion of patients with baseline hypertension (24% v. 25%) or diabetes (10% v. 10%).

**Figure 1:**
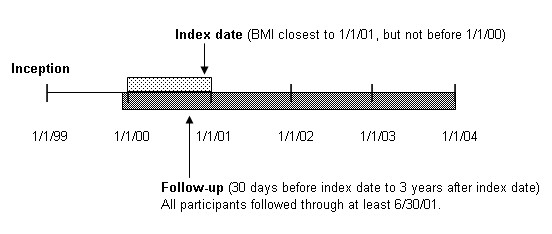
Study timeline, showing inception (1/1/99), range of index dates (1/1/00 to 12/31/00), and follow-up time period (30 days before index date to 3 years after index date).

We excluded 662 participants with diabetes mellitus or impaired glucose tolerance at baseline from blood glucose screening analysis. We excluded 1057 participants with hypertriglyceridemia, low HDL-cholesterol, or high LDL-cholesterol at baseline from lipid screening analysis. We defined the index date as BMI measurement closest to December 31, 2000 but not before January 1, 2000. This approach provided up to a 2-year window, January 1, 1999 to December 31, 2000, to assess baseline diabetes and lipid abnormalities. We defined follow-up as 30 days before index date through 3 years after index date. (see Figure 1)

All procedures were in accordance with the ethical standards for human subjects established by the Declaration of Helsinki. The institutional review board of Harvard Pilgrim Health Care approved the study protocols. Informed consent of individual patients was not necessary as this was a study of deidentified data.

### Measurements

From electronic medical records, we obtained height and weight, sex, race/ethnicity, and date of birth to calculate age at index date. We included a missing category for race/ethnicity because values were missing on 35% of patients. We also assessed baseline cardiovascular risk factors, including hypertension, diabetes mellitus, hypertriglyceridemia, low HDL-cholesterol, and high LDL-cholesterol, between January 1, 1999 to index date. (see Additional file 1)

We obtained laboratory data to assess the outcome measures, which were screening tests done during the follow-up period for blood glucose (random or fasting), triglycerides (fasting), and HDL-cholesterol (random or fasting) and LDL-cholesterol (fasting).

### Statistical Analysis

We assessed 3-year screening proportions by categories of BMI, age, sex, race/ethnicity, and baseline hypertension and (except for blood glucose screening) diabetes mellitus status. Next we assessed time-to-screening by multivariate proportional hazards models. We right-censored participants when membership in the health plan ended, at first date of diagnosis of medical condition causing weight loss (AIDS, cancer, malabsorption syndrome, or alcohol use disorder), or at first date of a cardiovascular event (congestive heart failure, unstable angina, myocardial infarction, coronary artery bypass grafting, percutaneous coronary intervention, coronary artery disease, ischemic stroke, visceral arterial disease, or peripheral arterial disease), whichever came first. Our primary model included BMI category, age category, sex and race/ethnicity. In subsequent models, we added baseline hypertension and diabetes mellitus as covariates.

Using parameter estimates from our multivariate models, we estimated predicted 3-year screening proportions among selected patients who were at low, moderate, high, and very-high risk for coronary heart disease and diabetes that we defined by levels of age, BMI, race/ethnicity, and baseline hypertension status, separately for males and females. For each combination of risk factors, we indicate whether published guidelines recommend screening for diabetes or dyslipidemia [4-7]. In this paper we show predicted screening proportions for 12 of the possible 160 combinations of risk factors, which we selected to show a wide variety of the possible combinations.

Using baseline characteristics, we also segregated patients in the cohort into two categories: those who should have been screened and those who did not need to be screened based on USPSTF guidelines [6,7]. Then, among those who should have been screened, we assessed 3-year screening rates and evaluated determinants of under-screening using multivariate proportional hazards models.

## Results

Among 5025 patients, mean (SD, range) age was 47.4 (15.6, 17.1 – 97.7) years; 3484 (69%) were female and 1541 (31%) were male. At baseline, 24%, 46%, 20%, and 10% of males and 46%, 29%, 14%, and 10% of females had a BMI of <25, 25–<30, 30–<35, and ≥35 kg/m^2^, respectively. Table 2 shows that increasing BMI category was associated with higher rates of baseline cardiovascular risk factors. For example, 18% of males in the lowest BMI category had baseline hypertension and rates rose monotonically to 48% in the highest BMI category. Baseline diabetes rates increased from 7% to 32%, hypertriglyceridemia rates increased from 6% to 20%, and low HDL cholesterol increased from 7% to 24% among males from lowest to highest BMI category. Men and women had different absolute rates of baseline cardiovascular risk factors, but similar increases across BMI categories.

**Table 2:** Baseline characteristics by BMI category and sex (N = 1541 males and N = 3484 females)

	**BMI (kg/m^2^)**							
	
	Males				Females			
	
	<25	25–<30	30–<35	≥35	<25	25–<30	30–<35	≥35
	(n = 374)	(n = 705)	(n = 315)	(n = 147)	(n = 1618)	(n = 1010)	(n = 500)	(n = 356)
	% of Subjects							
	
Age, years								
<35	26	15	10	11	34	24	20	22
35–44	21	21	22	27	27	23	23	24
45–54	26	27	31	33	19	22	20	29
55–64	12	16	16	14	7	11	15	14
≥65	15	21	20	16	12	20	23	12
Race/ethnicity								
Black	4	4	6	10	4	8	11	13
Hispanic	1	1	2	2	1	2	2	1
Other	10	5	2	1	8	6	4	1
White	38	52	53	43	56	53	52	50
Missing	48	39	38	44	31	31	31	35
Hypertension	18	30	44	48	10	24	34	38
Diabetes	7	12	22	32	3	6	18	22
Hypertriglyceridemia	6	17	23	20	3	11	19	18
Low HDL	7	17	20	24	4	14	24	22
High LDL	8	17	17	16	4	11	18	13

A total of 66% of patients had a glucose screening test (10% fasting and 90% random). Table 3 shows that compared with patients with BMI < 25 kg/m^2^, the adjusted hazard ratios (HR) for glucose screening were 1.2, 1.4, and 1.5 for patients with BMI 25–<30, 30–<35, and ≥35 kg/m^2^, respectively. Females were somewhat less likely to be screened than males (HR 0.9, [95% CI 0.8, 1.0]). Hispanic patients were more likely to be screened than whites (1.5 [1.1, 2.0]). Patients with higher age were more likely to be screened: HR 3.6 [3.1, 4.1] for patients ≥65 compared with patients <35 years). In a second model, further adjusted for baseline hypertension, we found that baseline hypertension was only a modest independent predictor of glucose screening (1.1 [1.0, 1.2]).

**Table 3:** Proportion screened and adjusted hazard ratios of blood glucose and lipid screening

	**Glucose (N = 4363)**				**Triglyceride (N = 3968)**		**HDL-C (N = 3968)**		**LDL-C (N = 3968)**	
	**N**	**Proportion Screened (%)**	**Adjusted HR (95% CI)**	**N**	**Proportion Screened (%)**	**Adjusted HR (95% CI)**	**Proportion Screened (%)**	**Adjusted HR (95% CI)**	**Proportion Screened (%)**	**Adjusted HR (95% CI)**

**Model 1.**										
Age (years)										
<35	1197	41%	1.0 (ref)	1150	19%	1.0 (ref)	34%	1.0 (ref)	18%	1.0 (ref)
35–44	1123	61%	1.6 (1.5, 1.8)	1067	36%	1.9 (1.6, 2.2)	53%	1.6 (1.4, 1.9)	34%	1.9 (1.6, 2.3)
45–54	1007	79%	2.7 (2.4, 3.0)	895	52%	3.0 (2.5, 3.5)	69%	2.5 (2.2, 2.8)	52%	3.1 (2.7, 3.7)
55–64	458	83%	3.0 (2.6, 3.4)	374	66%	4.1 (3.4, 4.9)	80%	3.3 (2.9, 3.9)	65%	4.3 (3.6, 5.2)
≥65	578	90%	3.6 (3.1, 4.1)	482	62%	4.1 (3.4, 4.9)	78%	3.3 (2.8, 3.8)	62%	4.3 (3.6, 5.2)
BMI (kg/m2)										
<25	1887	57%	1.0 (ref)	1799	32%	1.0 (ref)	49%	1.0 (ref)	31%	1.0 (ref)
25–<30	1485	70%	1.2 (1.1, 1.3)	1292	45%	1.3 (1.1, 1.4)	60%	1.1 (1.0, 1.2)	44%	1.3 (1.1, 1.4)
30–<35	632	76%	1.4 (1.2, 1.5)	538	51%	1.5 (1.3, 1.7)	65%	1.3 (1.1, 1.5)	49%	1.4 (1.2, 1.7)
≥35	359	75%	1.5 (1.4, 1.8)	339	57%	1.9 (1.6, 2.3)	68%	1.6 (1.4, 1.9)	55%	1.9 (1.6, 2.2)
Sex										
Male	1254	73%	1.0 (ref)	1120	54%	1.0 (ref)	67%	1.0 (ref)	52%	1.0 (ref)
Female	3109	63%	0.9 (0.8, 1.0)	2848	36%	0.7 (0.6, 0.8)	52%	0.8 (0.7, 0.8)	35%	0.7 (0.6, 0.8)
Race/ethnicity										
White	2288	65%	1.0 (ref)	2059	41%	1.0 (ref)	58%	1.0 (ref)	40%	1.0 (ref)
Black	267	64%	1.1 (0.9, 1.3)	256	37%	1.0 (0.8, 1.3)	54%	1.0 (0.9, 1.2)	36%	1.0 (0.8, 1.3)
Hispanic	67	70%	1.5 (1.1, 2.0)	64	31%	1.0 (0.7, 1.6)	52%	1.2 (0.8, 1.7)	30%	1.0 (0.6, 1.6)
Other	257	67%	1.3 (1.1, 1.6)	237	38%	1.1 (0.9, 1.3)	55%	1.0 (0.9, 1.3)	41%	1.1 (0.9, 1.3)
Missing	1484	66%	1.1 (1.0, 1.2)	1352	41%	1.1 (1.0, 1.2)	56%	1.0 (0.9, 1.1)	37%	1.1 (1.0, 1.2)
**Model 2. Model 1 + Diabetes**										
Diabetes										
No	--	--	--	3787	39%	1.0 (ref)	55%	1.0 (ref)	38%	1.0 (ref)
Yes	--	--	--	181	72%	1.5 (1.2, 1.8)	80%	1.3 (1.1, 1.6)	71%	1.4 (1.2, 1.7)
**Model 3. Model 1 + Hypertension**										
Hypertension										
No	3555	61%	1.0 (ref)	3285	36%	1.0 (ref)	53%	1.0 (ref)	35%	1.0 (ref)
Yes	808	85%	1.1 (1.0, 1.2)	683	61%	1.1 (1.0, 1.2)	76%	1.1 (1.0, 1.3)	60%	1.1 (0.9, 1.2)

CI = confidence interval

Overall, 41% of patients were screened for triglyceride level. As with glucose screening, triglyceride screening rose substantially across categories of BMI and age. (Table 3) Females were substantially less likely to be screened than males (0.7, [0.6, 0.8]). In two additional models, further adjusted for baseline diabetes and hypertension, we found that diabetes was a strong independent predictor of triglyceride screening (1.5 [1.2, 1.8]) whereas hypertension predicted only a modest increase in screening rates (1.1 [1.0, 1.2]).

The overall proportion screened of LDL-cholesterol (40%) was similar to that of triglyceride screening, but HDL-cholesterol showed a higher overall rate (57%). For both HDL- and LDL-cholesterol, as with triglyceride screening, higher BMI and age predicted higher screening rates, and female sex predicted lower rates. Likewise, baseline diabetes and hypertension predicted higher HDL-cholesterol screening rates to an extent similar to that of triglyceride screening. (Table 3)

Table 4 shows predicted 3-year screening proportions among low, moderate, high, and very-high risk patients, by sex, and contrasted these rates with the ADA, ATP III, and USPSTF recommendations [4-7]. For both diabetes and lipid screening, clinicians screened at least 80% of very-high risk persons, which we defined as 55–64 years old, BMI ≥ 35 kg/m^2^, non-white, with baseline hypertension. In contrast, clinicians screened persons that we deemed high risk somewhat less frequently than recommended. For example, ADA, ATP III, and USPSTF recommend both diabetes and lipid screening among patients 35–44 years old, BMI 30–<35 kg/m^2^, non-white, with baseline hypertension, but clinicians screened only 43% (LDL-cholesterol for females) to 83% (diabetes for males) of these patients. As expected, screening rates for low- and moderate-risk patients were lower still.

**Table 4:** Predicted 3-year screening proportions among selected low, moderate, high, and very-high risk patients, by sex, and whether published guidelines recommend screening for diabetes and dyslipidemia.

**Risk level**	**Age (years)**	**BMI (kg/m2)**	**Race/ethnicity**	**HTN**	**Sex**	**Glucose**	**Triglyceride**	**HDL-C**	**LDL-C**	**Screening recommended**			
										*ADA*	*ATP III*	*USPSTF*	

										*DM*	*Lipid*	*DM*	*Lipid*

Low	<35	<25	White	No	F	41%	18%	36%	17%	No	No	No	No
					M	44%	25%	44%	23%				
Moderate	<35	<25	Non-white	No	F	48%	19%	38%	18%	Yes	No	No	No
					M	52%	25%	46%	24%				
	35–44	25–<30	White	No	F	63%	37%	56%	36%	Yes	Yes	No	No
					M	67%	49%	66%	47%				Yes
High	35–44	30–<35	Non-white	Yes	F	79%	46%	67%	43%	Yes	Yes	Yes	Yes
					M	83%	58%	76%	55%				
	45–54	≥35	White	Yes	F	90%	70%	85%	68%	Yes	Yes	Yes	Yes
					M	92%	82%	92%	80%				
Very high	55–64	≥35	Non-white	Yes	F	95%	81%	93%	80%	Yes	Yes	Yes	Yes
					M	97%	91%	97%	89%				

BMI = body mass index. HTN = hypertension during the baseline time period. HDL-C = HDL-cholesterol. LDL-C = LDL-cholesterol. ADA = American Diabetes Association. ATP III = National Cholesterol Education Program. USPSTF = U.S. Preventive Services Task Force. DM = diabetes mellitus.

In a secondary analysis, we examined determinants of screening only among those for whom guidelines recommend screening. Based on USPSTF guidelines, 27% and 53% of patients should have been screened for diabetes and lipid disorders, respectively [5,7]. Among those who should have been screened for diabetes, 83% were screened. Among those who should have been screened for lipid disorders, the screening rate for LDL-cholesterol (55%) was similar to that of triglycerides (56%), but HDL-cholesterol showed a higher screening rate (71%). For both diabetes and lipid screening, increasing BMI was associated with higher rates of screening. Patients whose BMI was ≥ 35 were 41% more likely to be screened for diabetes than those whose BMI was <25 (HR 1.4, [95% CI 1.1, 1.7]); analogous hazard ratios for the other outcomes were 1.7 (1.4, 2.1) for triglycerides, 1.6 (1.3, 1.9) for HDL-cholesterol, and 1.7 (1.4, 2.0) for LDL-cholesterol. Older age was also associated with higher screening rates, with hazard ratios for those ≥ 65 years old ranging from 1.9 to 2.5 compared to those <35 years old. Women were screened at lower rates than men for lipid disorders (hazard ratios 0.8), but sex was not associated with diabetes screening rates (HR 1.1, [95% CI 0.95, 1.2]).

## Discussion

For both diabetes and lipid screening, we found that screening rates increased with BMI and age and were lower among females than males. We did not find that screening rates were lower among racial/ethnic minorities. The same patterns held whether we examined determinants of screening among cohort as a whole or among the subset for whom the USPSTF recommends screening.

Despite the fact that higher BMI, older age, and presence of hypertension (and diabetes) predicted appropriately higher screening rates, some absolute rates lagged behind recommendations. For diabetes and lipid screening, clinicians adequately screened very-high risk patients (at least 80% across screening tests). However, patients who were younger (35–44 years old) and less obese (BMI 30–<35 kg/m^2^), but still high enough risk to meet screening guidelines, were screened less often, 43% to 83% depending on the test and patient sex. Screening rates for the moderate-risk patients were lower still, however the evidence for screening is weaker. (Table 4)

We found that females were screened less than males across all risk factor levels. While females are at lower risk than males for developing CHD, screening guidelines do not differ by sex, except that male sex counts as a risk factor in determining optimal LDL-cholesterol level [6]. Also, USPSTF recommends lipid screening for males, but not females, who were in our moderate risk categories of 35–44 years old, BMI 25–<30 kg/m^2^, white, without baseline hypertension [7]. One possible explanation for underscreening among females is that women obtain some of their primary care from non-internal medicine providers, such as gynocologists, who may not routinely screen patients for diabetes and dyslipidemia. Other possible explanations are provider bias or failure to control for confounding factors such as education status or income level. Unfortunately, we did not have data to address these hypotheses.

Racial/ethnic minorities were not screened less than whites. In fact Hispanic persons had higher rates of glucose screening, which appears appropriate given that Hispanic Americans have higher prevalence of diabetes than whites [12].

We found that screening rates for triglyceride (41%) and LDL-cholesterol (40%) were lower than for glucose (66%) and HDL-cholesterol (57%). One possible explanation for lower triglyceride and LDL-cholesterol screening rates is that these tests require fasting blood samples for accurate measurement, whereas HDL-cholesterol and glucose do not. Another possible explanation is that clinicians were following USPSTF guidelines, which do not recommend for or against triglyceride measurement as part of routine screening for lipid disorders.

This study had several strengths. The sample size was relatively large, included males and females, and had some racial/ethnic diversity. In addition, the medical group practice employed fully electronic medical records throughout the study period, which we used in our analysis for physician diagnoses, procedures, medication use, laboratory results, and blood pressure and BMI measurements. Because patients included in our analysis were continuously enrolled in both an insurance plan and a medical group and had a BMI measurement, our results may not generalize to other populations.

Limitations of this study include lack of data on socioeconomic status. In addition, race/ethnicity was missing on 34% of patients. However, in a comparison of race data from the electronic medical records to member self-reports, it was found that blacks were only slightly more likely to be counted as missing (38% of blacks vs. 32% of whites) relative to whites (unpublished data, Adams AS). Therefore, we do not think that missing race is a bias. We were also missing data on two risk factors that ATP III recommends to assign LDL-cholesterol risk category, namely cigarette smoking and family history of premature coronary heart disease. The LDL-cholesterol goal for persons with multiple (at least two of five) risk factors is <130 mg/dL. We may have incorrectly used the goal <160 mg/dL instead of 130 for patients who smoked or had a family history of premature CHD, which would have reduce the number of patients excluded for high LDL-cholesterol at baseline. The lack of data on two of five risk factors could have biased screening rates in either direction. For example, we would expect a patient with an abnormal LDL level at baseline to get follow-up tests more often, which would falsely increase screening rates. On the other hand, a clinician may be waiting longer than the 3-year follow-up period to re-screen a patient with a normal baseline screening result, which would have falsely decreased screening rates. We did not examine blood concentrations of the risk factors, or evaluate the extent to which physicians appropriately treated, managed, and controlled patient risk factor levels after an abnormal screening result. Therefore, we could not assess the direction of LDL-cholesterol screening bias.

We could not estimate over-screening of low risk patients because we lacked information on risk factors that the guidelines prescribed. Also, while our follow-up period was 3 years, national guidelines recommend diabetes screening every three years and lipid screening every five years for low risk persons [4-7].

Although we excluded participants with diabetes mellitus or impaired glucose tolerance at baseline from blood glucose screening analysis, we could not completely distinguish between diabetes screening and diabetes case finding. For example, a patient with new onset polyuria but undiagnosed diabetes would be included in our glucose screening analysis.

## Conclusion

In summary, while patients with higher BMIs were more likely to be screened for both diabetes and dyslipidemia, a substantial portion of overweight and obese patients were not screened to the extent that national guidelines recommend. In addition, females appeared to be under-screened for these cardiometabolic risk factors. Future research should compare health outcomes among screened and unscreened individuals, and examine more detailed determinants of screening practices in medical care. Health plans and clinical groups could develop interventions to improve screening rates.

## Competing interests

None declared for JPF. SRS, KL, and MWG received grant support from Sanofi-Aventis. HC is employed as Director of Epidemiology in the US Medical Affairs Department of Sanofi-Aventis.

## Authors' contributions

SLR refined the study design, performed the statistical analysis and drafted the manuscript. JPF, KL, HC, and MWG conceived the study. MWG obtained funding. All authors made substantial contributions to design of the overall study, interpretation of data, and writing of the paper. All authors read and approved the final manuscript.

## Pre-publication history

The pre-publication history for this paper can be accessed here:



## Supplementary Material

Additional file 1 Definitions of baseline cardiovascular risk factors. The file includes definitions of baseline cardiovascular risk factors, including hypertension, diabetes mellitus, hypertriglyceridemia, low HDL-cholesterol, and high LDL-cholesterol. Click here for file

## References

[B1] Kuczmarski RJ, Carroll MD, Flegal KM, Troiano RP (1997). Varying body mass index cutoff points to describe overweight prevalence among U.S. adults: NHANES III (1988 to 1994). Obes Res.

[B2] Ford ES, Williamson DF, Lie S Weight change and diabetes incidence: findings from a national cohort of US adults. Am J Epidemiol.

[B3] Lipton RB, Liao Y, Cao G, Cooper RS, McGee D Determinants of incident non-insulin-dependent diabetes mellitus among blacks and whites in a national sample. The NHANES I Epidemiologic Follow-up Study. Am J Epidemiol.

[B4] (2002). The Expert Committee on the Diagnosis and Classification of Diabetes Mellitus. Diabetes Care.

[B5] Screening for Type 2 Diabetes Mellitus in Adults: Recommendations and Rationale. U.S. Preventive Services Task Force (USPSTF). http://www.ahrq.gov/clinic/3rduspstf/diabscr/diabetrr.htm.

[B6] (2001). Third Report of the National Cholesterol Education Panel (NCEP) Expert Panel on Detection, Evaluation, and Treatment of High Blood Cholesterol in Adults (Adult Treatment Panel or ATP III). NIH Publication No 01-3305.

[B7] (2001). Screening for Lipid Disorders: Recommendations and Rationale. Article originally in Am J Prev Med.

[B8] Ealovega MW, Tabaei BP, Brandle M, Burke R, Herman WH (2004). Opportunistic screening for diabetes in routine clinical practice. Diabetes Care.

[B9] Kern LM, Callahan MA, Brillon DJ, Vargas M, Mushlin AI Glucose testing and insufficient follow-up of abnormal results: a cohort study. BMC Health Serv Res.

[B10] Ahluwalia IB, Mack KA, Murphy W, Mokdad AH, Bales VS (2003). State-specific prevalence of selected chronic disease-related characteristics – Behavioral Risk Factor Surveillance System, 2001. MMWR Surveill Summ.

[B11] Henriksen K, Battles JB, Marks ES, Lewin DI, Chan for the HMO Research Network CERT Safety Investigators (2005). Advances in patient safety: from research to implementation Concepts and methodology.

[B12] (2005). National Institute of Diabetes and Digestive and Kidney Diseases. National Diabetes Statistics fact sheet: general information and national estimates on diabetes in the United States, 2005.

