# Supplementary material for: Diabetes and lipid screening among patients in primary care: A cohort study
Source: BMC Health Serv Res. 2008 Jan 30;8:25. doi: 10.1186/1472-6963-8-25 (PMC2266727; doi:10.1186/1472-6963-8-25)
Supplement: Additional file 1 — Definitions of baseline cardiovascular risk factors. The file includes definitions of baseline cardiovascular risk factors, including hypertension, diabetes mellitus, hypertriglyceridemia, low HDL-cholesterol, and high LDL-cholesterol. [file 1472-6963-8-25-S1.doc]

Additional file 1. Definitions of baseline risk factors.

| Hypertension [1]  - A single blood pressure measurement with systolic blood pressure ≥140 mmHg and/or diastolic blood pressure ≥90 in combination with filling a prescription for an antihypertensive medication within 3 months of the blood pressure measurement or - Two or more outpatient diagnoses of hypertension ≥30 days apart (ICD-9 = 401.xx) or - One inpatient diagnosis of hypertension (ICD-9 = 401.xx) or - One outpatient diagnosis of hypertension (ICD-9 = 401.xx) in combination with filling a prescription for an antihypertensive medication within 3 months of the diagnosis |
| --- |
| **Diabetes mellitus** [2]   - Any fasting glucose measurement ≥126 or - Any random glucose measurement ≥200 or - One inpatient diagnosis of diabetes mellitus (ICD-9 = 250.xx or 250.xx) or - One or more outpatient diagnosis of diabetes mellitus (ICD-9 = 250.xx or 250.xx) in combination with filling a prescription for diabetic therapy within 3 months of the diagnosis or - Two or more outpatient diagnoses of diabetes mellitus ≥30 days apart (ICD-9 = 250.xx or 250.xx) or - Filling a prescription for insulin or sulfonylurea preparation. |
| **Hypertriglyceredemia** [3]   - Any fasting triglyceride measurement ≥150 mg/dL or - Filling a prescription for either a Fibric Acid Derivative or Nicotinic Acid Derivative. |
| **Low HDL cholesterol** [3]   - Filling a prescription for either a Fibric Acid Derivative or a Nicotinic Acid Derivative or - Any HDL measurement <40 (if male) or <50 (if female) |
| High LDL cholesterol [3]  - Filling a prescription for a Statin or - Any LDL measurement >100 mg/dL if CHD or CHD risk equivalents (transient ischemic attack, ischemic stroke, peripheral arterial disease, coronary artery disease, diabetes mellitus, unstable angina, myocardial infarction or coronary artery bypass grafting) or - Any LDL measurement >130 if at least 2 of 3 risk factors*:   - Hypertension   - Low HDL cholesterol   - Age (men >45, women >55)   or   - Any LDL measurement >160 if 0 – 1 risk factors   *We do not have data on these 2 additional risk factors: cigarette smoking, family history of premature CHD. |

ICD-9 = International Classification of Diseases, 9th Revision.

**References**

1. Chobanian AV, Bakris GL, Black HR, Cushman WC, Green LA, Izzo JL Jr, Jones DW, Materson BJ, Oparil S, Wright JT Jr, Roccella EJ. The Seventh Report of the Joint National Committee on Prevention, Detection, Evaluation, and Treatment of High Blood Pressure: the JNC 7 report.
   JAMA. 2003 May 21;289(19):2560-72
2. The Expert Committee on the Diagnosis and Classification of Diabetes Mellitus (2002) Diabetes Care 25, S5–S20.
3. Third Report of the National Cholesterol Education Panel (NCEP) Expert Panel on Detection, Evaluation, and Treatment of High Blood Cholesterol in Adults (Adult Treatment Panel or ATP III). NIH Publication No. 01-3305. May 2001.
